# Comparing self-regulation strategies among adult learners from Poland, Serbia, Slovakia, and the Czech Republic

**DOI:** 10.3389/fpsyg.2024.1382989

**Published:** 2024-11-26

**Authors:** Jitka Vaculíková

**Affiliations:** Research Centre of the Faculty of Humanities, Tomas Bata University in Zlín, Zlín, Czechia

**Keywords:** self-regulation, self-control, decision making, goal orientation, adults

## Abstract

An active and constructive process whereby individuals possess the ability to develop, implement, and flexibly maintain planned behavior in order to achieve a desired achievement goal is referred to as self-regulation. The aim of the present study is to examine the factorial structure and psychometric properties of the Self-Regulation Questionnaire, validated in the Czech educational context (SRQ-CZ). The other target is to identify any usage of self-regulation strategies which may differ as nationality, gender, age, education, and internal motivation to learn come into play. A total of 1,711 adult learners from Poland, Serbia, Slovakia, and the Czech Republic who were enrolled in a formal secondary or higher education system pursuing professions in the field of humanities, social and health care sciences participated in this study. A measurement-invariant four-factor model was obtained across all countries (min. CFI, TLI = 0.918, and 0.902, max. RMSEA = 0.059; *ω* between 0.625 and 0.838, and Cronbach’s *α* between 0.622 and 0.837), including the Impulse Control, Goal Orientation, Self-Direction, and Decision Making subscales. Generally, the study confirmed a gradual increase in scores that measure positive self-regulatory qualities (Goal Orientation and Decision Making) and a decrease in unfavorable self-regulatory qualities (Impulse Control and Self-Direction) with higher age, education, and motivation, with no gender differences reported within countries. Moreover, scores on Goal Orientation and Decision Making dominated between countries. In conclusion, the SRQ-CZ demonstrated its suitability for cross-national comparisons, and personal characteristics appear to be important factors that distinguish those with high and low self-regulation.

## 1 Introduction

Contemporary society, through its challenges and pressures on its members, fosters the need for an individual’s ability to self-direct their learning and behavior and adjust to the rapidly changing environment. The competence of self-regulation is a key element in trying to be a successful student in adolescence, or a competitive employee who easily deals with the requests of the labor market and the increased demand for lifelong learning in later life (UNESCO, 2019, 2021). Therefore, receiving fewer directions or formative assessments from the external environment requires self-regulatory skills. In general, self-regulation focuses on the manner in which individuals orient their behaviors, cognition, and affect in order to achieve their goals (Brown et al., 1999). Self-regulated individuals are active participants who initiate and direct their efforts and capabilities for acquiring knowledge and skills, take responsibility for their actions, and maintain motivation (Zimmerman, 1990, 2000).

Self-regulation capacity in adult education is generally considered an individual responsibility and is recognized worldwide due to its positive impact on health, proper adjustment, and physical and mental well-being (Briki, 2016, 2017; Hennessy et al., 2020). However, self-regulation in higher education can differ among specific groups according to their cultural background (McInerney, 2008; Mclnerney and King, 2018; Salili et al., 2001). Although the body of literature in the field has significantly grown during the last decades (e.g., Jakešová et al., 2016; Vaculíková et al., 2022; Virtanen et al., 2015), research targeting adults’ self-regulation strategies in higher education has been less frequent, primarily in the case of culture-specific non-Western Anglo educational context.

Moreover, there is an inconsistency among scientists about which phases are involved in self-regulation. Kanfer (1970) and Carver and Scheier (1982) have proposed a three-phase theory of self-regulation that includes self-monitoring, self-evaluation, and self-reinforcement. Miller and Brown (1991) elaborated on Kanfer’s model and extended the number of phases involved in self-regulation to seven, including information input, self-monitoring, triggering change, searching for options, planning, implementation, and assessing a plan’s effectiveness. Based on this theory, the authors developed the Self-Regulation Questionnaire (SRQ; Brown et al., 1999) widely used and tested across a variety of samples and life domains, and reported several factorial solutions (Gavora et al., 2015; Pichardo et al., 2014, p. 1018; Potgieter and Botha, 2009; Vaculíková et al., 2022).

In addition, two other versions of this research instrument were developed. Carey et al. (2004) provided preliminary psychometric evidence from the SRQ and reported a single factor structure including 31 items, titled the Short Self-Regulation Questionnaire (SSRQ). Neal and Carey (2005) proposed two distinct factors, impulse control and goal setting. In summary, the SRQ lacks appropriate data fit and has demonstrated unstable factorial structure resulting in diverse numbers of components. Despite those limitations, SRQ belongs to a palette of widely adopted research instruments that allow comparisons of self-regulatory abilities of adult learners.

### 1.1 Personal characteristics and self-regulation

In an early study Zimmerman and Martinez-Pons (1990) proposed that boys surpassed girls in the use of self-regulatory strategies, including self-monitoring, goal setting, planning, and structuring of their study environment, while other researchers (Pajares, 2002; Vandevelde et al., 2013) found that males tended to show less self-regulating behavior than females. Hosseini-Kamkar and Morton (2014) reported in their review study that, paradoxically, gender differences in self-regulation were more consistently reported in children than adults. A few studies have suggested that females are less impulsive than males during the fertile phases of the menstrual cycle (Pine and Fletcher, 2011; Khaighobadi and Stevens, 2013). Accordingly, van Tetering et al. (2020) presented clear differences in self-regulation as perceived by 13–16-year-old mid-adolescents. In this age period, females reached higher levels of self-control and self-monitoring.

Education level is believed to be a strong factor that influences self-regulation. For example, in two longitudinal, prospective studies of middle school students, Duckworth et al. (2012) found that over time, self-control predicted grades better than IQ. Similarly, achieved level of education proved to be an important factor distinguishing those with high and low impulsivity in a representative sample of Czech adults and university students (Jakešová et al., 2016). Moreover, self-regulation, commonly researched in connection with learning, could be described as a multi-component process (Zimmerman and Risemberg, 1997). From this point of view, self-regulation includes cognitive, metacognitive, behavioral, emotional, and motivational aspects of learning (Boekaerts, 2011; Pintrich and De Groot, 1990; Pintrich, 2000). Problem-behavior theories suggest that motivation and regulation may operate interactively (Ernst and Fudge, 2009), and individuals’ levels of self-regulation might differ in relation with their intrinsic motivations. Pintrich and Duncan (1993) reported that those students with higher levels of intrinsic orientation and task value tended to report higher levels of cognitive and self-regulatory strategy use. Students who had positive motivational beliefs (higher levels of intrinsic orientation, task value, and self-efficacy) were more likely to regulate their cognition by planning, monitoring, and regulating their use of study strategies. This connection would help to explain why some intelligent students perform poorly in their studies. Building on situational and contextual approaches activation of motivational beliefs differs in terms of study context and subject area. Leaving the perception of motivation as a personality trait, students may respond differently to challenges in the academic environment (Boekaerts, 2001; Volet, 2001).

As briefly mentioned, self-regulation is an important individual ability that strongly affects one’s actions and behavior. Over the last 50 years, self-regulation in various forms has been investigated in educational, clinical, and health psychology, social cognitive theory, and adjacent disciplines; the emergent conclusion is that the ability to control attentional, emotional, or behavioral impulses in the service of personally valued goals and standards (Duckworth and Carlson, 2015) may protect individuals against serious health risks (Carey et al., 1990; Chassin and DeLucia, 1996) and self-regulation failure is shown to lead to poor academic (or work) performance (Baumeister and Heatherton, 1996; Howell and Watson, 2007). Senécal et al. (1995) found that self-regulation alone explains almost a third of the variance in academic procrastination that involves more than poor time management skills or trait laziness. Moreover, self-regulation is not only seen as a goal-oriented process that controls inappropriate behavior but also as the capacity for planning to initiate appropriate behavior at any stage of life (Wehmeyer and Shogren, 2017). Some evidence shows that high levels of self-regulatory capacities promoting the development of identity achievement play a vital role in learners’ psychological well-being in general by lending a sense of continuity to one’s life (Hofer et al., 2011).

In sum, capturing and explicating individual differences in the use of self-regulation strategies and their connections is an important goal of current research on prospective human functioning. However, there is a lack of any valid and stable instrument measuring the self-regulation of adult learners outside the Anglosphere or findings describing the influence of personal characteristics. Therefore, the main aims of this study were to assess the factor structure and psychometric properties of the SRQ-CZ (Gavora et al., 2015) in a multi-cultural sample of European adult learners, and to identify the usage of self-regulation strategies according to personal characteristics, including the most relevant: nationality, gender, age, level of higher education, and motivation.

### 1.2 The present study

This study examines self-regulation strategies from a cross-cultural perspective. Specifically, we focused on adult learners enrolled in a formal secondary or higher education system as measured by the SRQ-CZ (Gavora et al., 2015). Based on the development of self-regulation embedded in a cultural context that prioritizes a specific model of agency, self-regulated strategies are assumed to differ cross-culturally. In this research, culture refers to subjective point of view, including values, beliefs, and traditions that foster self-regulatory skills through social collaboration, modeling, guidance and feedback (Mclnerney and King, 2018).

From the starting point of this pilot study of a long-term research investigation, four European countries were purposefully selected: Poland, Serbia, Slovakia, and the Czech Republic. We chose these countries for several reasons. At first, they share similar cultural, linguistic and political backgrounds, climate and geographic location. Second, based on the varieties of capitalism approach and welfare regime classifications (Esping-Andersen, 1998), they belong to post-socialist embedded neoliberal welfare state regime (WSR), except Serbia, which belongs to post-socialist Western Balkan WSR, characterized by common socialist past and coordinated market economies that significantly shaped their access to the labor markets, requirements for adult education and training with different learning systems. The Slovaks and the Czechs are the closest nationalities that formed a joint federation for more than 74 years, followed by sensible and non-violent disintegration after 1992.

Based on these assumptions, our aim is to extend previous psychometric evaluations of the SRQ-CZ by determining the factor structure and internal consistency of the SRQ-CZ (Gavora et al., 2015), and at the same time, to identify the usage of self-regulation strategies among adults. Four sub-aims of the study are to evaluate (1) the construct validity of the SRQ-CZ, (2) its measurement invariance across selected countries, as well as gender, age, education, and motivation groups, (3) the reliability of its factors among compared groups of respondents and (4) to identify any usage of self-regulation strategies. The rationale for our aims lies in the fact that the original SRQ (Brown et al., 1999) was previously used mainly in English-speaking (e.g., Carey et al., 2004; Neal and Carey, 2005) and Spanish-speaking countries (e.g., Pichardo et al., 2014, p. 1018; Potgieter and Botha, 2009), and therefore deserves closer investigation in culturally diverse context.

Following up with the discussion presented in previous sections, in this study, we shall relate the four dimensions of the self-regulation of behavior (see the Results section) in four samples (i.e., Polish, Serbian, Slovak and Czech) to five variables: nationality, gender, age, level of higher education, and motivation. Accordingly, we have formulated the following research questions (RQ1-2), along with related general hypotheses (H1-2):

RQ1: To what extent is variation in scores on self-regulation strategies dependent on the country where the respondents live?

H1: The scores on self-regulation strategies will differ within and between countries. In this regard, we proposed the following sub-hypotheses:

H1a: Scores on self-regulation strategies in Slovakia and the Czech Republic will share a similar pattern.

H1b: Scores on self-regulation strategies in Poland and Serbia will share a similar pattern.

RQ2: How do the scores on self-regulation strategies differ when gender, age, education, and internal motivation to learn are investigated?

H2: The scores on self-regulation strategies will differ by personal characteristics in all samples. In this regard, we proposed the following sub-hypotheses:

H2a: Scores on self-regulatory strategies of females will be higher than those of males in all samples (i.e., females better self-regulate their behavior).

H2b: Scores on self-regulatory strategies of older adults will be higher than those of younger adults in all samples (i.e., older adults better self-regulate their behavior).

H2c: Scores on self-regulatory strategies of higher-educated adults will be higher than those of lower-educated adults in all samples (i.e., adults with higher education better self-regulate their behavior).

H2d: Scores on self-regulatory strategies of adults with a high level of intrinsic motivation (above the group average) towards education will be higher than those of adults with a low level in all samples (i.e., adults with high intrinsic motivation better self-regulate their behavior).

## 2 Materials and methods

### 2.1 Sample and procedure

Adult learners of higher education in the surveyed countries aged between 18 and 64 constituted the target population for this study. Surveys were administrated separately in all countries from January to March 2022 by national research teams using a self-completed internet surveying technique or by a specialized agency using the Computer Assisted Web Interviewing method (CAWI). The invitation to participate in the research was distributed across countries and targeted schools, respecting their geographical breakdown. Data collection was disrupted by a wave of the Covid-19 pandemic, and the research team had to compromise on the original plan for a representative data set of the adult population in key demographic characteristics such as age, gender, education, and size of residence. Instead, data collection was based on a convenient sample of adult learners (aged between 18 and 64) enrolled in formal education. Respondents voluntarily participated in data collection, respecting that all questionnaire items were set as mandatory, so there were no missing values in the data. Participating countries obtained ethical approval of the study procedures from the institutional ethics committee and the data collection and data analysis in this study have followed ethical principles of research, respecting the ICC/ESOMAR International Code (ESOMAR, 2016).

The present study comprises 1,711 European adult learners from Poland (*n* = 276), Serbia (*n* = 410), Slovakia (*n* = 511), and the Czech Republic (*n* = 514) enrolled in a formal secondary or higher education system. Ages ranged from 18 (Poland) to 61 years (Serbia), with an average age of 25.4 years (*SD* = 7.3). Female adults considerably outnumbered male adults including 1,356 (79.3%) females (*M*age = 25.1 years; *SD* = 7) and 355 (20.7%) males (*M*age = 26.36 years, *SD* = 7.9). In total, the majority of adult learners (1,294, 75.6%) had attained an upper secondary to a first tertiary degree in the form of bachelor’s degree (ISCED 3–6). 426 (24.9%) of adult learners had attained ISCED 7–8 covering programs designed to provide advanced academic, professional or research qualification and knowledge at one of the leading government-funded universities across selected countries. Specialization of the programs offered by the secondary or tertiary institutions included studies in humanities, social and health care sciences. Therefore, graduates pursue professions in state administration, self-government and the non-profit sector, or represent professionals employed by the hospitals and home care, work in counselling, or institutions for the handicapped and retirement homes. Lastly, intrinsic motivation divided all samples, with almost half of adults falling below or above average, including 803 (46.9%) adults with low and 908 (53.1%) adults with high intrinsic motivation. The detailed descriptive statistics in each sample and pooled sample are presented in Table 1.

**Table 1:** Univariate description of the samples.

Variables	Poland (*n* = 276)	Serbia (*n* = 410)	Slovakia (*n* = 511)	Czech Republic (*n* = 514)	Pooled sample (*n* = 1,711)
			*n* (%)		
Gender					
Male	23 (8.3)	105 (25.6)	98 (19.2)	129 (25.1)	355 (20.7)
Female	253 (91.7)	305 (74.4)	413 (80.8)	385 (74.9)	1,356 (79.3)
Age					
18–26 years	176 (63.8)	237 (57.8)	455 (89)	426 (82.9)	1,294 (75.6)
27–61 years	100 (36.2)	173 (42.2)	56 (11)	88 (17.1)	417 (24.4)
Education					
ISCED 3–6	210 (76.1)	286 (69.8)	349 (68.3)	440 (85.6)	1,285 (75.1)
ISCED 7–8	66 (23.9)	124 (30.2)	162 (31.7)	74 (14.4)	426 (24.9)
Motivation					
Low	140 (50.7)	185 (45.1)	248 (48.5)	230 (44.7)	803 (46.9)
High	136 (49.3)	225 (54.9)	263 (51.5)	284 (55.3)	908 (53.1)

### 2.2 Instrument

#### 2.2.1 Self-regulation

The original SRQ (Brown et al., 1999) is a self-reporting instrument designed to assess self-regulation capacity in seven phases, covered by 63 items with response options on a five-point Likert scale ranging from “strongly disagree” to “strongly agree” along equal intervals. These phases represent the steps needed for effective self-regulation that involves monitoring of behavior, comparing it with reference values and introducing adjustments if necessary. Moreover, it reflects the ability to receive relevant information (i.e., “I usually keep track of my progress toward my goals”), evaluate and contrast it to one’s norms (i.e., “My behavior is similar to that of my friends”), trigger change (i.e., “I am willing to consider other ways of doing things”), search for options (i.e., “If I wanted to change, I am confident that I could do it”), formulate a plan (i.e., “Once I have a goal, I can usually plan how to reach it”), implement the plan (i.e., “I can stick to a plan that’s working well”), and assess its effectiveness (i.e., “I feel bad when I do not meet my goals”).

The psychometric properties of the original SRQ were evaluated on diverse samples, including a community sample (Aubrey et al., 1994), college and university students (Carey et al., 2004; Gavora et al., 2015; Neal and Carey, 2005; Potgieter and Botha, 2009), and clients with drug issues (Bukhtawer et al., 2014). However, the factor structure, including seven phases (or dimensions) of self-regulation, was not empirically supported by any of these studies.

A previous attempt to confirm a seven-phase theory within Central Europe also failed. Instead, the Czech version of the SRQ, labeled SRQ-CZ (Gavora et al., 2015), yielded a model with four factors: Impulse Control (8 items), Goal Orientation (5 items), Self-Direction (7 items), and Decision Making (7 items). The second application of the SRQ-CZ across adult learners included in this study (i.e., from Poland, Serbia, Slovakia, and the Czech Republic) yielded a three-factor model as the result of exploratory and confirmatory factor analyses: Impulse Control and Self-Direction merged into one distinct factor, while Decision Making and Goal Orientation comprised the other two (Vaculíková et al., 2022). In contrast to these previous studies, we are interested in revealing patterns in adult learners’ self-regulation strategies that would indicate invariants and differences among samples in four culturally close European countries. However, the construct validity and reliability of the SRQ-CZ were again evaluated within the present dataset. For a detailed overview of the instrument’s preparation and validation, see Vaculíková et al. (2022).

#### 2.2.2 Personal characteristics

Gender was assessed with a dichotomous variable asking respondents whether they were male or female. Age was computed based on the respondent’s year of birth. In case of measurement invariance, age was further assigned to two categories: 18–26 and 27–61 years old. Educational background was measured by an ordinal-response item that was divided to represent upper secondary to tertiary education (ISCED 3–6) and Master’s or doctoral or equivalent level (ISCED 7–8).

Intrinsic motivation towards education was measured by the Academic Motivation Scale (AMS-28, Vallerand et al., 1993). The original AMS-28, based on self-determination theory, includes seven subtypes of motivation that fall along a continuum of relative autonomy: three types of intrinsic motivation, three types of extrinsic motivation, and amotivation evenly divided into 28 items on a 7-point Likert scale ranging from 1 “does not correspond at all” to 7 “corresponds exactly.” Although AMS-28 has been extensively used in the field of higher education (Howard et al., 2017), there is a lack of valid instruments outside the English-speaking (Vallerand et al., 1993) or Spanish-speaking populations (Stover et al., 2012), except for Ardeńska et al. (2019) and Kubiatko (2018) who supported the use of the Polish and Slovak versions of the AMS in university and high school students, respectively. More recently, Kočvarová et al. (2024) confirmed the underlying linguistic and cultural consensus of the original seven-factor structure in four countries (i.e., Poland, Serbia, Slovakia, and the Czech Republic) and extended its use in higher education in other Eastern European countries like Hungary, Latvia, or Romania.

Based on AMS-28, the three types of intrinsic motivation were included in this study: intrinsic motivation towards knowledge or knowing (4 items), intrinsic motivation towards accomplishment (4 items), and intrinsic motivation towards experiencing stimulation (4 items). Means were considered low for respondents who scored below the mean and high for respondents who fell within the mean and above the country’s group average.

### 2.3 Data analysis

The presented statistical methods were used to analyze cross-cultural data. A confirmatory factor analysis (CFA) was performed to evaluate a four-factor structure, based on the comparative fit index (CFI), Tucker-Lewis index (TLI), and root mean square error of approximation (RMSEA). Acceptable cut-offs for the CFI and TLI indices were set at a value higher than 0.90, with a value of ≥0.95 as good, and the RMSEA cut-off point was set as acceptable at a value of 0.08, with a value of ≤0.06 as good (Hu and Bentler, 1999). We concentrated on the solid structural validity of items that had factor loadings of ≥0.50 (Costello and Osborne, 2005). In addition, we examined configural (a qualitatively invariant measurement pattern of factors; loadings and intercepts freely estimated), metric (a quantitatively invariant measurement model of factors; loadings equal and intercepts freely estimated), and scalar (the invariant mean levels of item intercepts; loadings as well as intercepts equal across groups) measurement invariance (i.e., no, partial, full) across country (four nationalities), gender (male and female), age (18–26 years and 27–61 years old), education (ISCED 3–6 and ISCED 7–8), and motivation (below and above the mean), with ΔCFI ≤0.01 and increased RMSEA by not more than 0.015 as signs of invariance (Chen, 2007). In other words, the higher the level of invariance, the stricter the degree of conformity and the higher the degree of comparability of the tested model across groups.

Intercorrelations of subscales were computed with a Spearman’s *rho* of 0.30 considered as a moderate correlation, and an *rho* of ≥0.50 as a large correlation (Cohen, 1988). Reliability coefficients were assessed with McDonald’s *ω* and Cronbach’s *α*, with a threshold of acceptability at ≥0.60 indicating an acceptable level of reliability (Taber, 2018). Kolmogorov–Smirnov and Shapiro–Wilk tests were used with nonparametric data. A mean difference analysis of the self-regulation within the samples was assessed with the Friedman test. The Kruskal-Wallis *H* test and Mann–Whitney *U* test were used for a comparison of two or several independent samples. The descriptive and multivariate analyses were conducted in IBM SPSS Statistics 29. IBM SPSS AMOS 29 Graphics was used to calculate CFA and MI. Moreover, we used JASP 0.18.3.0 for the calculation of McDonald’s *ω*.

## 3 Results

### 3.1 Construct validity and reliability of the SRQ-CZ in the samples

The CFA of a four-factor structure of the SRQ-CZ, in all countries, did not support a good model fit (CFI = 0.829, TLI = 0.811, RMSEA = 0.065), nor there was an acceptable fit in individual countries (min. CFI, TLI = 0.779, and 0.756, max. RMSEA = 0.077). Notably, low factor loadings of several items appeared across all factors. In such cases, the items at issue must be avoided (Ximénez, 2009). Therefore, to avoid the presence of weak factors, defined by small loading sizes, we decided to perform CFAs only with factor loadings of ≥0.50 (Costello and Osborne, 2005). In all countries, the CFA showed a good model fit (min. CFI, TLI = 0.918, and 0.902, max. RMSEA = 0.059) (see Supplementary Table S1). Although factor loadings did not always exceed a lower bound of 0.50 within revised structure (see Table 2), a purified 18-item four-factor model confirmed a solid structural validity. An overview of the original English version and the four language permutations of the questionnaire (i.e., Polish, Serbian, Slovak and Czech) are included in Supplementary Table S6.

**Table 2:** Summary CFA results of the four-factor model across all countries.

Factor*	No.	Item wording	Min. loading^a^	Max. loading^b^	Average loading^c^
IC	3	I get easily distracted from my plans.	0.62	0.73	0.68
	5	It’s hard for me to see anything helpful about changing my ways.	0.45	0.68	0.59
	6	When it comes to deciding about a change, I feel overwhelmed by the choices.	0.47	0.62	0.54
	7	I have trouble following through with things once I’ve made up my mind to do something.	0.73	0.82	0.78
	9	I can come up with lots of ways to change, but it’s hard for me to decide which one to use.	0.57	0.63	0.61
	27	I give up quickly.	0.65	0.71	0.69
GO	12	I have personal standards and try to live up to them.	0.61	0.79	0.70
	13	I am set in my ways.	0.75	0.83	0.78
	19	I have rules that I stick by no matter what.	0.63	0.87	0.78
SD	4	I do not notice the effects of my actions until it’s too late.	0.61	0.73	0.65
	8	I do not seem to learn from my mistakes.	0.53	0.62	0.59
	15	I have a hard time setting goals for myself.	0.56	0.63	0.60
	21	Often, I do not notice what I’m doing until someone calls it to my attention.	0.41	0.55	0.49
DM	14	As soon as I see a problem or challenge, I start looking for possible solutions.	0.64	0.71	0.68
	17	As soon as I see things aren’t going right, I want to do something about it.	0.51	0.66	0.60
	18	There is usually more than one way to accomplish something.	0.52	0.56	0.53
	20	I can usually find several different possibilities when I want to change something.	0.61	0.70	0.66
	23	I’m good at finding different ways to get what I want.	0.55	0.75	0.64
Summary of loadings (18 items)			0.41	0.87	0.64

* = “IC” indicates Impulse Control, “GO” Goal Orientation, “SD” Self-Direction, and “DM” Decision Making; No., number of items. ^a^ Lowest observed factor loading across countries. ^b^ Highest observed factor loading across countries. ^c^ Average factor loading across countries.

The average correlation between factors was medium or rather small (Table 3), showing the strongest relationship between Impulse Control and Self-Direction. The internal consistency, as measured by McDonald’s *ω*, ranged between 0.625 and 0.838, and Cronbach’s *α* ranged between 0.622 and 0.837 (see Supplementary Table S2), suggesting an acceptable reliability of individual factors across all countries. However, as mentioned by Taber (2018), simply reaching threshold level does not guarantee instrument quality. Therefore, we provided evidence of dimensionality supported by factor analysis, and in addition, using multi-group CFA, we examined whether the factor structure was measurement invariant across subgroups.

**Table 3:** Summary correlation results of the four-factor model across all countries.

Factor*	IC			GO			SD		
	Min.^a^	Max.^b^	Average^c^	Min.^a^	Max.^b^	Average^c^	Min.^a^	Max.^b^	Average^c^
GO	−0.13	−0.28	−0.23						
SD	0.65	0.74	0.54	−0.20	−0.31	−0.24			
DM	−0.26	−0.41	−0.34	0.36	0.46	0.41	−0.22	−0.38	−0.31

* = “IC” indicates Impulse Control, “GO” Goal Orientation, “SD” Self-Direction, and “DM” Decision Making; correlations are significant at the 0.01 level. ^a^ Lowest observed correlation coefficient across countries. ^b^ Highest observed correlation coefficient across countries. ^c^ Average correlation coefficient across countries.

### 3.2 Measurement invariance of the SRQ-CZ

Table 4 shows that, in all countries, constraining the factor loadings (metric invariance), means, and intercepts (scaler invariance) to be the same across groups (by gender, age, education, and motivation) did not substantially deteriorate model fit (max. ΔCFI = 0.005, max. ΔRMSEA = 0.001), excepting the country invariance established at a lower metric level (ΔCFI = 0.008, ΔRMSEA = 0.000). Although the obtained results can be considered satisfactory (full and partial invariance), we also addressed the question of how to increase the level of measurement invariance across countries up to scalar level. Comparison of parameter estimates of individual items among countries, as well as inspection of modification indices and any partial changes to the model did not bring the required conclusion.

**Table 4:** Goodness of fit of invariance tests (*n* = 1,711).

Grouping variable	Model fit			Change in model fit	
	CFI	TLI	RMSEA	ΔCFI	ΔRMSEA
Country invariance					
Configural	0.936	0.924	0.027		
Metric	0.928	0.921	0.027	0.008	0.000
Scalar	0.865	0.862	0.036	0.063	−0.009
Gender invariance					
Configural	0.941	0.930	0.036		
Metric	0.941	0.934	0.035	0.000	−0.004
Scalar	0.939	0.934	0.035	0.002	0.000
Age invariance					
Configural	0.941	0.930	0.036		
Metric	0.942	0.934	0.035	−0.001	0.001
Scalar	0.937	0.932	0.035	0.005	0.000
Education invariance					
Configural	0.940	0.929	0.036		
Metric	0.940	0.932	0.035	0.000	0.001
Scalar	0.939	0.935	0.034	0.001	0.001
Motivation invariance					
Configural	0.936	0.924	0.036		
Metric	0.935	0.927	0.035	0.001	0.001
Scalar	0.931	0.926	0.036	0.004	−0.001

All changes in model fit are significant at the 0.001 level.

According to the differences among the values of CFI and RMSEA, gender and age invariance was established within each country (see Supplementary Table S3). However, gender invariance was not established in the Polish sample, nor age invariance in Serbia. Education and motivation measurement invariance was established in all countries except Serbia, which did not reach a good model fit in either measurement invariance test, and the Czech Republic, in the case of motivational invariance (see Supplementary Table S4).

### 3.3 Self-regulation in the samples

The results were expressed as mean scores that were compared across factors and samples (Table 5), with all differences being statistically significant (regarding hypothesis H1). Overall, adult learners scored above the midpoint of the scale that measured positive self-regulatory qualities (Goal Orientation and Decision Making) and lower on those scales that indicate unfavorable self-regulation behavior (Impulse Control and Self-Direction). This indicated that adult learners have a relatively good ability to self-regulate their behavior. Between country groups, Goal Orientation and Decision Making were most prevalent in Serbian sample and Czech adults reached the highest scores on Impulse Control and Self-Direction when compared to other countries. Within each country, Slovaks and Czechs rated their Goal Orientation as the highest and Self-Direction as the lowest when compared with the rest of the self-regulatory strategies (supporting hypothesis H1a). Adult learners from Poland and Serbia rated their use of Decision Making highest, and similarly to other countries, Self-Direction as the lowest than their usage of other self-regulatory strategies (supporting hypothesis H1b) (see Table 5).

**Table 5:** Mean ratings of the self-regulation subscales within the samples.

	IC	GO	SD	DM	*p*
Samples	*M* (*SD*)	*M* (*SD*)	*M* (*SD*)	*M* (*SD*)	
Poland	2.69 (0.86)	3.54 (0.90)	2.44 (0.75)	3.89 (0.65)	**<0.001**
Serbia	2.58 (0.89)	3.84 (0.82)	2.31 (0.85)	3.99 (0.66)	**<0.001**
Slovakia	2.72 (0.83)	3.83 (0.88)	2.47 (0.84)	3.64 (0.69)	**<0.001**
Czech Republic	2.93 (0.82)	3.72 (0.85)	2.80 (0.86)	3.55 (0.70)	**<0.001**
*p*	**<0.001**	**<0.001**	**<0.001**	**<0.001**	

“IC” indicates Impulse Control, “GO” Goal Orientation, “SD” Self-Direction, “DM” Decision Making, and “SR” Self-Regulation; M = mean and SD = standard deviation on 5-point Likert scale; significant values are highlighted in bold.

### 3.4 Personal characteristics associated with self-regulation

#### 3.4.1 Gender

It was assumed that gender would make a significant difference for self-regulation strategies, as reported in the literature, although researchers have analyzed diverse self-regulatory components and samples (Liu et al., 2021; Pajares, 2002; Zimmerman and Martinez-Pons, 1990). However, this assumption was not confirmed in the present investigation (rejecting hypothesis H2a). Although females had higher scores than males in all samples except the Czech one, those differences were not significant (Table 6). The same non-significant results predominated when investigating individual factors separately (not shown in the table).

**Table 6:** Mean ratings of the self-regulation strategy by personal characteristics of the samples.

Self-regulation		Poland	Serbia	Slovakia	Czech Republic	*p*
		*M* (*SD*)	*M* (*SD*)	*M* (*SD*)	*M* (*SD*)	
Gender	Male	3.09 (0.34)	3.18 (0.41)	3.16 (0.40)	3.30 (0.42)	**=0.01**
	Female	3.15 (0.40)	3.18 (0.41)	3.17 (0.39)	3.24 (0.46)	**=0.05**
	*p*	0.469	0.937	0.711	0.076	
Age	18–26 years	3.19 (0.40)	3.19 (0.42)	3.17 (0.40)	3.23 (0.44)	0.148
	27–61 years	3.06 (0.38)	3.15 (0.39)	3.08 (0.37)	3.34 (0.48)	**<0.001**
	*p*	**<0.01**	0.328	0.186	0.112	
Education	ISCED 3–6	3.18 (0.40)	3.20 (0.42)	3.15 (0.41)	3.24 (0.44)	**<0.05**
	ISCED 7–8	3.02 (0.37)	3.13 (0.37)	3.20 (0.35)	3.35 (0.48)	**<0.001**
	*p*	**=0.001**	0.207	0.083	0.187	
Motivation	Low	3.12 (0.40)	3.15 (0.43)	3.12 (0.42)	3.17 (0.44)	0.184
	High	3.16 (0.39)	3.20 (0.39)	3.21 (0.36)	3.31 (0.45)	**<0.01**
	*p*	0.541	0.516	**<0.05**	**=0.01**	

M, mean; SD, standard deviation on 5-point Likert scale; significant values are highlighted in bold.

#### 3.4.2 Age

Because age is an important variable associated with personality growth, cognitive and metacognitive functioning (Murman, 2015; Siegel and Castel, 2019), and task performance (Czaja et al., 2001), we were interested to know how younger and older adults rate their self-regulation strategies (Table 6). In this study, age made a significant difference only in the self-regulation strategies rated by Polish adults (supporting hypothesis H2b in the Polish sample). More specifically, we documented a gradual increase in age when scores measured positive self-regulatory qualities (Goal Orientation and Decision Making) and a decrease in unfavorable self-regulatory qualities (Impulse Control and Self-Direction). For more details, see Supplementary Table S5.

#### 3.4.3 Education

An increase in self-regulation due to more demanding and complex learning tasks requiring more skill or effort to achieve academic success was expected in adults with higher education (Table 6). Similarly, only Polish adults gave a significantly different rating of self-regulation use (supporting hypothesis H2c in the Polish sample) with a gradual decrease in unfavorable self-regulatory qualities (Impulse Control and Self-Direction) with higher educational achievement (Supplementary Table S5).

#### 3.4.4 Motivation

In addition, self-regulation is a goal-driven activity, with self-motivation beliefs being a crucial component of many self-regulation models of self-regulated learning (i.e., Boekaerts, 2011; Zimmerman, 2000). Therefore, we were interested to find out whether the level of intrinsic motivation towards education differentiates the usage of self-regulatory strategies. This assumption was confirmed only in part (Table 6). In all countries, adults with high motivation (above the group average) had higher scores of self-regulation strategies than their counterparts. However, only the samples from Slovakia and the Czech Republic showed significant differences (supporting hypothesis H2d in the Slovak and Czech samples). More specifically, those adults who reached above the mean intrinsic motivation also reported higher levels of desirable self-regulatory strategies (Goal Orientation and Decision Making), and lower levels of Impulse Control. Moreover, intrinsic motivation made significant differences in Self-Direction, however, only in the Slovak sample. Slovak adults with high intrinsic motivation reported a lower level of unfavorable self-regulation strategy in the case of Self-Direction (Supplementary Table S5).

## 4 Discussion

The present cross-sectional study is developed in line with comparative research approaches (Berry et al., 2011), which aim to detect, analyze, and explain differences across cultures while taking into account a host of challenges. More specifically, this study addresses the discrepancy of findings regarding the relations between self-regulation, understood as individuals’ ability to act according to an internal plan to achieve personal goals, and investigates the influence of personal characteristics, as highlighted in the current literature (Liu et al., 2021; Martinez-Lopez et al., 2017). Therefore, one purpose of this study is to identify similarities and differences in self-regulation strategies used by adult learners from four European countries, from the perspective of four personal characteristics, using a valid and reliable instrument.

Evidence from 1,711 adult learners showed that the SRQ-CZ, validated in the Czech educational context, has good psychometric properties within an international context. The CFA confirmed a good structural validity of the scale across the pooled sample and individual countries. With proper fit indices, the final SRQ-CZ used in this study was shorter than the previous Czech version (Gavora et al., 2015; Vaculíková et al., 2022), which is a validated version of the original SRQ (Brown et al., 1999). More specifically, all countries used in this study showed a good structural validity by means of the good model fit of a four-factor model and high factor loadings of 18 items that were retrieved from the set of 27 items (Gavora et al., 2015). Similarly, the first round of SRQ-CZ validation found that the number of items can be reduced to well-fitting 22 items (Vaculíková et al., 2022). Chen and Lin (2018), while validating the Short Form Self-Regulation Questionnaire (SSRQ) in the Taiwanese educational context, as well as Motamed-Jahromi et al. (2022) among Iranian community-dwelling older adults, found that the number of items could be refined, and a shorter questionnaire was obtained. Likewise, Pichardo et al. (2014, 2018) showed goodness of fit with the four-factor model, with a reduced number of items obtained from the original 63-item SRQ instrument. It seems that the decrease in items compared to the original version of the instrument may be related to cultural, environmental, or population-based differences in the samples that were involved in the research.

Within this study, the internal consistency of the items was good in all countries, suggesting that the scale provides reliable scores. On average (i.e., in the pooled sample), the factor structure of the SRQ-CZ was measurement invariant at a metric level across the country (four groups), and measurement invariant at a scalar level across gender, age, education, and motivation (two groups per each variable), i.e., variables that were further compared across the pooled sample. This implies that the instrument measures the same underlying latent construct for males and females, older (27–61 years old) and younger adults (18–26 years old), adults with a bachelor’s or higher educational degree, and adults with a perceived high or low motivation to learn. However, more attention should be paid to the case of metric invariance when testing country-related groups. More specifically, it indicates that constraining the loadings across countries does not significantly affect the model fit (metric invariance). Still, at the same time, it does not empirically support our assumption that differences in the latent construct capture all mean differences in the shared variance of the items (scalar invariance).

Based on separate measurement invariance testing for predefined groups mentioned above within each country, measurement invariance led to satisfactory results in all samples except gender invariance in the Polish sample and motivational invariance in the Czech Republic. In line with previous research (Vaculíková et al., 2022), the lack of model fit appeared to be most serious in Serbia. While the Serbian sample did not vary from other country-divided samples used in this study, the same question recalls how specific Serbian self-regulation is compared to other countries. A new question arises, namely whether the construct validity was not affected by response biases due to the social desirability of Serbian respondents. However, the scales were evaluated as internally consistent across all countries, including Serbia, and therefore we decided to compare self-regulation strategies among all samples from adult learners, except Serbia. For further investigation, we would recommend including a representative sample of the Serbian population to analyze the psychometric properties of the instrument and to identify its subscales more precisely.

In this study, we used the SRQ-CZ (Gavora et al., 2015), which provides a proxy for four components that are important for proper self-regulation: Impulse Control, Self-Direction, Decision Making, and Goal Orientation. It should be noted that this part of the study represents the second round of the SRQ-CZ validation, using the same data but a different research strategy, which led us to slightly different results. On this basis, all the factors form separate subscales, as reported in the pioneering work of Gavora et al. (2015) on a sample of Czech university students. Those findings highlight that the same data can have more than one alternative model fit (Poldrack, 2019), and that a different perspective can be worthy of investigation.

The other aim of this study was to examine the self-regulated strategies in adult education across four European countries (regarding RQ1). The somewhat lower scores on subscales that reflect unfavorable self-regulatory behavior, including the low ability to manage a task to completion or decisions about changes, and the low ability to control one’s impulses when performing actions, were expected. On the contrary, the high scores of a pooled sample of adult learners on favorable self-regulatory subscales, including Goal Orientation and Decision Making, signify the good perceived ability of adult learners to plan their actions, set personal goals, and monitor their accomplishment. Such results are not surprising, since all samples included adult learners currently enrolled in a formal secondary or further higher education system, who have already entered adulthood with all its charms and challenges, and due to the intensive effort in learning regulation and training for their current or future jobs, their daily tasks involve self-control, rational planning, and acting.

Notably, a within-country comparison revealed that Slovak and Czech adult learners rated their use of Goal Orientation highest and Self-Direction as the lowest when compared with the rest of the self-regulatory strategies. On the other hand, adult learners from Poland and Serbia rated their Decision Making higher than other self-regulatory strategies, which includes a good self-regulatory ability to look for possible solutions or ways to get what they want; they also rated Self-Direction as the lowest. Therefore, we accepted both proposed hypotheses (H1a-b) regarding the impact of country of residence on perceived usage levels of self-regulatory strategies.

It shows that demographically and historically close nationalities that have experienced a government and history similar to Czechoslovakia are rather set in their ways with personal rules that they stick by no matter what. For a long time in their history, they did not have the opportunity to make any changes, which may still have an effect on their self-concepts, perceptions, and behavior, leading them to be more stable in their personal standards. This form of behavior is still characteristic of the mainstream public education sector where the present shows some traces of the past. Although positive shifts have occurred in the formulation of educational legislation, management, financing, and the curriculum, teacher professionalization and autonomy leading to partial stabilization and modernization, there are still barriers in the educational sector. Real systemic reform reaching the intermediate and micro-levels of the educational system has not been implemented yet (Greger and Walterová, 2007).

At the same time, educational psychologists could argue that other variables also play a significant role in shaping one’s self-regulation. However, higher education forms a significant part of adult learner’s lives, and other high-relevance variables (nationality, gender, age, education and motivation) were also utilized in this study (regarding RQ2). As concerns personal characteristics associated with self-regulation, our assumption was that females would outperform males in setting their goals and managing their decisions and actions. In other words, we expected that females better self-regulate their behavior; however, this hypothesis was not confirmed (H2a). Moreover, we documented a gradual increase in scores that measure positive self-regulatory qualities (Goal Orientation and Decision Making) and a decrease in unfavorable self-regulatory qualities (Impulse Control and Self-Direction) with higher age, education, and motivation in all countries, but only the Polish (age and education), Slovak, and Czech (motivation) mean differences reached the level of significance (hypotheses H2b-d were not accepted in all countries).

Demonstrating that personal characteristics and level of motivation do matter in self-regulation in higher education also has important implications while designing new or refreshing ongoing learning environments or interventions. It is important to create situations promoting a positive study atmosphere and active knowledge construction, developing more functional self-regulatory skills of personal goal-setting and decision-making, and minimizing those unfavorable self-regulatory strategies. To do so, it is appropriate to follow learners’ self-regulation, and their success expectations, evaluate preferences, and monitor the effectiveness of different teaching approaches. However, teachers play a crucial role in promoting of their students’ self-regulation practices. Exploring the interplay of their self-regulated competences as learners and as agents of self-regulation, i.e., how aspects of teachers’ competences impact their self-regulation instruction in classrooms, deserves current attention (Brenner, 2022; Karlen et al., 2020; Liu et al., 2019).

### 4.1 Strengths and limitations

The strengths of this study include the validation of the SRQ-CZ in adult learners, the use of separate samples from European countries, and the investigation of personal characteristics across self-regulatory subscales. There are, however, several limitations. While using cross-cultural data, we have assessed self-regulation using a self-reported instrument and without using an objective tool. As such, the reported mean scores may be over- or under-estimated due to socially desirability or retrospective biases in reporting or errors of memory, i.e., confounds in participants’ ability to recall experiences. Similarly, self-reports were not context-specific, rather they measure general self-regulatory skills. However, introducing confirmatory and comparative results, we decided to use general-level outcomes for adult learners coming from several nationalities and cultures.

Regarding the samples, the adult learners in this study reached a higher-than-average level of education and were enrolled in formal secondary or higher education systems. Therefore, their self-regulation abilities, as well as their motivation to learn, may vary from the target population. As described within the sample section of this study, female adults outnumbered male adults. However, females form the majority of students in humanities, social and health care sciences, and therefore reflect the proportion of the target population of students from the Faculty of Humanities and Faculty of Social Sciences included in this study. Based on the homogeneous nature of students’ specialization, we did not research differences concerning the faculties, programs, or subjects. Moreover, the cross-sectional design of the study precludes the possibility of assessing the predictive validity or test–retest reliability of the subscales. Considering these limitations, using longitudinal data and including more validation analyses, such as convergent and discriminant validity, is warranted in order to extend our knowledge about the psychometric properties of the SRQ-CZ.

To increase the level of country-related measurement invariance from metric to scalar, the three options for future data analysis appear to be beneficial (Putnick and Bornstein, 2016): sequentially release or add item intercept constraints and retesting the model until a partially invariant model is achieved (search for the source of noninvariance), purify the items pool with noninvariant intercepts and retest invariance models, or assume that the construct is no-invariant. Perhaps tests of a large number of groups (>2) rarely appear in the literature because many groups involved in analysis are more likely to detect nonconformity of results; complex models force researchers to research fewer groups, or non-significant studies sequestered in file drawers for no tolerance for null results (for the file drawer problem, see Rosenthal, 1979). Moreover, measurement invariance could also be tested separately for all possible pairs of the four countries. However, these invariant combinations would not support our intention to compare self-regulation strategies among all samples within a single test.

As for further research investigation, more research on the mathematical and practical implications of invariance testing few versus many groups is needed. In addition, it is perhaps time to develop alternative measurements outside Western research, rather than assuming their valid and reliable universality (Mclnerney and King, 2018). On this basis, there is a clear need for a cross-sectional and longitudinal approach to determine structural and developmental trends in adults’ self-regulation strategies across and beyond Europe in which new models of self-regulation and associated measures can be used as a diagnostic and research (and more indigenous) instrument.

## 5 Conclusion

This study highlights the importance of self-regulatory skills, which have been especially crucial during the recent long period of online learning, and could become standard in the future. Therefore, the self-regulation abilities of individuals of any age or specialization will be a hot topic. The theoretical significance and empirical contribution of this research study includes detailed information on the validation of the SRQ-CZ and the extension of conflicting findings regarding differences in self-regulation in higher education settings according to most relevant personal characteristics (i.e., gender, age, education, and motivation) across four European countries.

The presented results are important for three reasons. First, we offer evidence of mean differences in the generic ability to self-regulate behavior across four nationalities and four main personal characteristics, under the umbrella of a single-study investigation. Second, the presented findings strengthen our understanding of how self-regulatory abilities are utilized outside the Western and English-speaking countries. The results are based on heretofore neglected, demographically and historically similar European countries. Third, considering all limitations, we demonstrate the suitability of the SRQ-CZ for cross-national comparisons of self-regulation strategies, newly extended across the European context. Moreover, we have confirmed that Impulse Control, Goal Orientation, Self-Direction, and Decision Making are important in the self-regulation of behavior; however, they have a contrasting character.

## Data Availability

The raw data supporting the conclusions of this article will be made available by the authors, without undue reservation.
