# Supplementary material for: Comparing self-regulation strategies among adult learners from Poland, Serbia, Slovakia, and the Czech Republic
Source: Front Psychol. 2024 Nov 26;15:1382989. doi: 10.3389/fpsyg.2024.1382989 (PMC11628270; doi:10.3389/fpsyg.2024.1382989)
Supplement: Supplementary file 1 [file Data_Sheet_1.pdf]

Supplementary Table 1. CFA results on the pooled sample and subsamples divided by country

	Model fit				Factor loadings																	
Subsample	No.	CFI	TLI	RMSEA	i3	i5	i6	i7	i9	i27	i12	i13	i19	i4	i8	i15	i21	i14	i17	i18	i20	i23
Pooled sample	18	.945	.935	.049	**.64**	**.57**	**.54**	**.79**	**.62**	**.70**	**.71**	**.79**	**.74**	**.66**	**.60**	**.61**	**.51**	**.70**	**.62**	**.57**	**.64**	**.64**
Poland	18	.941	.931	.052	**.70**	**.68**	**.62**	**.73**	**.57**	**.71**	**.61**	**.83**	**.79**	**.61**	**.53**	**.56**	.41	**.67**	**.51**	**.56**	**.70**	**.75**
Serbia	18	.918	.902	.059	**.73**	.45	**.52**	**.77**	**.63**	**.69**	**.64**	**.77**	**.63**	**.73**	**.61**	**.63**	.46	**.64**	**.66**	**.52**	**.61**	**.55**
Slovakia	18	.957	.949	.043	**.62**	**.64**	.47	**.82**	**.62**	**.65**	**.77**	**.75**	**.87**	**.63**	**.59**	**.58**	**.55**	**.68**	**.63**	**.52**	**.64**	**.55**
Czech Republic	18	.927	.914	.059	**.66**	**.58**	**.55**	**.80**	**.62**	**.70**	**.79**	**.76**	**.83**	**.63**	**.62**	**.62**	**.54**	**.71**	**.61**	**.53**	**.67**	**.71**

Note: No. = number of items; i = item; boldface denotes factor loadings of ≥ .50.

Supplementary Table 2. Reliability coefficients across subsamples

Subsample	Factor	McDonald's ω Point estimate	95% CI lower bound	95% CI upper bound	Cronbach´s α Point estimate	95% CI lower bound	95% CI upper bound
Country							
Poland	IC	.831	.800	.862	.830	.797	.859
(*n* = 276)	GO	.797	.756	.839	.779	.731	.820
	SD	.625	.552	.697	.622	.543	.690
	DM	.779	.738	.820	.775	.729	.814
Serbia	IC	.808	.779	.837	.801	.770	.829
(*n* = 410)	GO	.725	.678	.771	.712	.662	.756
	SD	.704	.658	.750	.697	.645	.742
	DM	.739	.698	.779	.736	.693	.774
Slovakia	IC	.807	.782	.833	.802	.774	.828
(*n* = 511)	GO	.838	.814	.862	.837	.811	.860
	SD	.677	.631	.723	.677	.628	.720
	DM	.750	.715	.784	.745	.709	.778
Czech Republic	IC	.817	.793	.841	.812	.786	.836
(*n* = 514)	GO	.835	.810	.860	.835	.809	.858
	SD	.697	.654	.740	.695	.650	.736
	DM	.787	.758	.816	.781	.750	.809
Pooled sample	IC	.812	.798	.825	.808	.794	.822
(*n* = 1,711)	GO	.793	.776	.810	.790	.772	.806
	SD	.689	.665	.713	.688	.664	.712
	DM	.774	.757	.791	.771	.753	.788

Note: "IC" indicates Impulse Control (6 items), "GO" Goal Orientation (3 items), "SD" Self-Direction (4 items), and "DM" Decision Making (5 items).

Supplementary Table 3. Gender and age measurement invariance tests by country

	Model fit			Change in model fit			Model fit			Change in model fit	
Gender invariance	CFI	TLI	RMSEA	ΔCFI	ΔRMSEA	Age invariance	CFI	TLI	RMSEA	ΔCFI	ΔRMSEA
Poland						Poland					
Configural	.897	.878	.050			Configural	.941	.930	.036		
Metric	.891	.878	.050	.006	.000	Metric	.941	.933	.035	.000	.001
Scalar	.894	.886	.048	-.003	.002	Scalar	.947	.944	.032	-.006	.003
Serbia						Serbia					
Configural	.918	.902	.042			Configural	.913	.897	.043		
Metric	.916	.906	.041	.002	.001	Metric	.912	.901	.042	.001	.001
Scalar	.913	.907	.041	.003	.000	Scalar	.898	.891	.044	.014	-.002
Slovakia						Slovakia					
Configural	.940	.929	.036			Configural	.943	.932	.035		
Metric	.941	.934	.035	-.001	.001	Metric	.942	.935	.035	.001	.000
Scalar	.940	.936	.035	.001	.000	Scalar	.941	.937	.034	.001	.001
Czech Republic						Czech Republic					
Configural	.928	.914	.041			Configural	.919	.904	.044		
Metric	.928	.919	.040	.000	.001	Metric	.919	.909	.043	.000	.001
Scalar	.924	.918	.041	.004	-.001	Scalar	.916	.910	.043	.003	.000

Supplementary Table 4. Education and motivation measurement invariance tests by country

Education invariance	Model fit			Change in model fit		Motivation invariance	Model fit			Change in model fit	
	CFI	TLI	RMSEA	ΔCFI	ΔRMSEA		CFI	TLI	RMSEA	ΔCFI	ΔRMSEA
Poland						Poland					
Configural	.920	.905	.043			Configural	.929	.916	.039		
Metric	.925	.916	.040	-.005	.003	Metric	.928	.919	.038	.001	.001
Scalar	.928	.923	.039	-.003	.001	Scalar	.923	.917	.039	.005	-.001
Serbia						Serbia					
Configural	.910	.893	.043			Configural	.892	.872	.046		
Metric	.903	.891	.044	.007	-.001	Metric	.890	.876	.045	.002	.001
Scalar	.894	.886	.045	.009	-.001	Scalar	.879	.871	.046	.011	.005
Slovakia						Slovakia					
Configural	.946	.936	.034			Configural	.947	.937	.033		
Metric	.947	.940	.033	-.001	.001	Metric	.945	.938	.033	.002	.000
Scalar	.946	.943	.033	.001	.000	Scalar	.941	.937	.033	.004	.000
Czech Republic						Czech Republic					
Configural	.922	.907	.043			Configural	.914	.898	.044		
Metric	.921	.911	.042	.001	.001	Metric	.910	.898	.044	.004	.000
Scalar	.922	.916	.041	-.001	.001	Scalar	.908	.901	.044	.002	.000

Supplementary Table 5. Personal characteristics significantly associated with individual self-regulation strategies by country

Country	Grouping variable	Test	IC	GO	SD	DM
Poland	Age	Mann-Whitney *U* test	6079.500	7588.000	6217.000	7427.500
		Wilcoxon W	11129.500	23164.000	11267.000	23003.500
		Z	-4.276	-1.915	-4.073	-2.163
		p	**.000**	**.050**	**.000**	**.031**
	Education	Mann-Whitney *U* test	4917.000	6529.500	5380.000	6614.000
		Wilcoxon W	7128.000	28684.500	7591.000	28769.000
		Z	-3.565	-.713	-2.754	-.561
		p	**.000**	.476	**.006**	.575
Slovakia	Motivation	Mann-Whitney *U* test	25254.500	24830.500	28626.500	18845.500
		Wilcoxon W	59970.500	55706.500	63342.500	49721.500
		Z	-4.419	-4.705	-2.400	-8.286
		p	**.000**	**.000**	**.016**	**.000**
Czech Republic	Motivation	Mann-Whitney *U* test	27561.000	25495.500	31031.000	19846.000
		Wilcoxon W	68031.000	52060.500	71501.000	46411.000
		Z	-3.052	-4.313	-.977	-7.684
		p	**.002**	**.000**	.329	**.000**

Note: "IC" indicates Impulse Control, "GO" Goal Orientation, "SD" Self-Direction, and "DM" Decision Making; significant values are highlighted in bold.

Supplementary Table 6. Original English and four language (Polish, Serbian, Slovak and Czech) permutations of the selected variables.

Variable	Description
**English version**	
Gender	*Are you?*<br>- Male<br>- Female
Age	*How old are you?*<br>- The exact age in the format YYYY (years)
Education	*What is your highest achieved education?*<br>- Elementary or unfinished education<br>- Vocational or high school without baccalaureate<br>- Vocational with baccalaureate<br>- High school with baccalaureate<br>- College<br>- University – bachelor’s degree<br>- University – master’s degree<br>- University – doctoral degree
Intrinsic motivation (AMS-28)	*Using the scale below, indicate to what extent each of the following items presently corresponds to one of the reasons why you participate in continuing education. Please, use 1 “does not correspond at all” to 7 “corresponds exactly.”*<br>- Because I experience pleasure and satisfaction while learning new things.<br>- For the intense feelings I experience when I am communicating my own ideas to others.<br>- For the pleasure I experience while surpassing myself in my learning.<br>- For the pleasure I experience when I discover new things never seen before.<br>- For the pleasure that I experience when I read something interesting.<br>- For the pleasure that I experience while I am surpassing myself in one of my personal accomplishments.<br>- For the pleasure that I experience in broadening my knowledge about subjects which appeal to me.<br>- For the pleasure that I experience when I feel completely absorbed by certain ideas.<br>- For the satisfaction I feel when I am in the process of accomplishing difficult tasks.<br>- Because my studies allow me to continue to learn about many things that interest me.<br>- For the "high" feeling that I experience while reading about various interesting subjects.<br>- Because it allows me to experience a personal satisfaction in my quest for self-development.

Self-regulation (SRQ-CZ)	*Please indicate to what extent each of the following items presently corresponds to your behavior. Please, use 1 "strongly disagree" to 5 "strongly agree."*<br>- I usually keep track of my progress toward my goals.<br>- I have trouble making up my mind about things.<br>- I get easily distracted from my plans.<br>- I don't notice the effects of my actions until it's too late.<br>- It's hard for me to see anything helpful about changing my ways.<br>- When it comes to deciding about a change, I feel overwhelmed by the choices.<br>- I have trouble following through with things once I've made up my mind to do something.<br>- I don't seem to learn from my mistakes.<br>- I can come up with lots of ways to change, but it's hard for me to decide which one to use.<br>- I can stick to a plan that's working well.<br>- I usually only have to make a mistake one time in order to learn from it.<br>- I have personal standards, and try to live up to them.<br>- I am set in my ways.<br>- As soon as I see a problem or challenge, I start looking for possible solutions.<br>- I have a hard time setting goals for myself.<br>- When I'm trying to change something, I pay a lot of attention to how I'm doing.<br>- As soon as I see things aren't going right I want to do something about it.<br>- There is usually more than one way to accomplish something.<br>- I have rules that I stick by no matter what.<br>- I can usually find several different possibilities when I want to change<br>- Often I don't notice what I'm doing until someone calls it to my attention.<br>- Usually I see the need to change before others do.<br>- I'm good at finding different ways to get what I want.<br>- I usually think before I act.<br>- I learn from my mistakes.<br>- I know how I want to be.<br>- I give up quickly.
**Polish version**	
Płeć	*Czy jesteś?*<br>- Mężczyzna<br>- Kobieta

Wiek	*Ile masz lat?*<br>- Dokładny wiek w formacie RRRR (lata)
Edukacja	*Jakie jest Twoje najwyższe wykształcenie?*<br>- Edukacja podstawowa lub niedokończona<br>- Szkoła zawodowa lub średnia bez matury<br>- Zawodowe z maturą<br>- Liceum z maturą<br>- Szkoła Wyższa<br>- Uniwersytet – licencjat<br>- Uniwersytet – tytuł magistra<br>- Uniwersytet – stopień doktora
Wewnętrzna motywacja (AMS-28)	*Korzystając z poniższej skali, wskaż, w jakim stopniu każda z poniższych pozycji odpowiada obecnie jednemu z powodów, dla których uczestniczysz w kształceniu ustawicznym. Proszę użyć skali od 1 „w ogóle nie odpowiada” do 7 „odpowiada dokładnie”.*<br>- Ponieważ doświadczam przyjemności i satysfakcji podczas uczenia się nowych rzeczy.<br>- Podczas przekazywania własnych pomysłów innym osobom doświadczam intensywnych uczuć.<br>- Dla przyjemności której doświadczam, udoskonalając siebie podczas studiów.<br>- Dla przyjemności, której doświadczam, kiedy odkrywam nowe, nigdy wcześniej nieznane rzeczy.<br>- Dla przyjemności, której doświadczam, kiedy czytam opracowania interesujących autorów.<br>- Dla przyjemności, jakiej doświadczam, kiedy udoskonalam siebie.<br>- Dla przyjemności, której doświadczam poszerzając swoją wiedzę na tematy, które mi się podobają.<br>- Dla przyjemności, której doświadczam, kiedy czuję się całkowicie pochłonięty, gdy czytam opracowania naukowe.<br>- Dla satysfakcji jaką odczuwam, gdy jestem w trakcie realizacji trudnych działań akademickich.<br>- Ponieważ moje studia pozwalają mi kontynuować naukę dotyczącą wielu kwestii, które mnie interesują.<br>- Gdy czytam różne interesujące naukowe opracowania, doświadczam uczyć wyższych.<br>- Ponieważ studia pozwalają mi doświadczyć osobistej satysfakcji w dążeniu do doskonałości w nauce.
Samoregulacja (SRQ-CZ)	*Proszę wskazać, w jakim stopniu każdy z poniższych elementów odpowiada obecnie Twojemu zachowaniu. Proszę użyć skali od 1 „zdecydowanie się nie zgadzam” do 5 „zdecydowanie się zgadzam”.*<br>- Zwykle śledzę swoje postępy w realizacji moich celów.<br>- Trudno mi podjąć decyzję.<br>- Łatwo odwracam uwagę od moich planów.<br>- Efekty swoich działań zauważam dopiero, gdy jest już za późno.

- Trudno mi dostrzec coś pomocnego w zmianie mojego postępowania.
- Kiedy podejmuję decyzję o zmianie, czuję się przytłoczony wyborami.
- Mam problemy z realizacją zadań, gdy już coś postanowię.
- Wygląda na to, że nie uczę się na błędach.
- Mogę wymyślić wiele sposobów na zmianę, ale ciężko mi się zdecydować, który z nich zastosować.
- Potrafię trzymać się planu, który działa dobrze.
- Zazwyczaj wystarczy, że popełnię błąd tylko raz, żeby wyciągnąć z niego naukę.
- Mam osobiste standardy i staram się ich przestrzegać.
- Jestem nastawiony na swoje.
- Gdy tylko widzę problem lub wyzwanie, zaczynam szukać możliwych rozwiązań.
- Trudno mi wyznaczać sobie cele.
- Kiedy próbuję coś zmienić, dużą uwagę zwracam na to, jak sobie radzę.
- Gdy tylko widzę, że coś nie idzie dobrze, chcę coś z tym zrobić.
- Zazwyczaj istnieje więcej niż jeden sposób osiągnięcia czegoś.
- Mam zasady, których się trzymam bez względu na wszystko.
- Kiedy chcę się zmienić, zazwyczaj znajduję kilka różnych możliwości.
- Często nie zauważam tego, co robię, dopóki ktoś nie zwróci mi na to uwagi.
- Zwykle widzę potrzebę zmiany, zanim zrobią to inni.
- Jestem dobry w znajdywaniu różnych sposobów na osiągnięcie tego, czego chcę.
- Zwykle myślę, zanim coś zrobię.
- Uczę się na swoich błędach.
- Wiem, jaki chcę być.
- Szybko się poddaję.

Serbian vesion

Pol	*Jesi li?*<br>- Muški<br>- Žensko
Starost	*Koliko imaš godina?*<br>- Tačna starost u formatu GGGG (godine)
Edukacija	*Koje je vaše najviše dostignuto obrazovanje?*<br>- Osnovno ili nezavršeno obrazovanje<br>- Stručna ili srednja škola bez mature<br>- Stručno sa maturom

	- Srednja škola sa maturom<br>- Koledž<br>- Univerzitet – diploma<br>- Univerzitet – master<br>- Univerzitet – doktorat
Unutrašnja motivacija (AMS-28)	*Koristeći donju skalu, označite u kojoj meri svaka od sledećih stavki trenutno odgovara jednom od razloga zašto učestvujete u kontinuiranom obrazovanju. Molimo, koristite 1 „uopšte ne odgovara“ do 7 „tačno odgovara“.*<br>- Jer osećam ispunjenost i zadovoljstvo dok učim nove stvari.<br>- Zbog intenzivnih osećanja koje doživljavam kada prenosim svoje ideje drugima.<br>- Iz zadovoljstva koje doživljavam nadmašujući sebe na studijama.<br>- Iz zadovoljstva koje doživljavam kada otkrijem nove stvari koje nikada ranije nisam video.<br>- Iz zadovoljstva koje doživljavam kada čitam zanimljive autore.<br>- Iz zadovoljstva koje doživljavam dok nadmašujem sebe u jednom od svojih ličnih dostignuća.<br>- Iz zadovoljstva koje doživljavam šireći svoje znanje o predmetima koji me zanimaju.<br>- Iz zadovoljstva koje doživljavam kada se osećam potpuno zaokupljeno onim što su pojedini autori napisali.<br>- Iz zadovoljstva koje osećam kada sam u procesu obavljanja teških akademskih aktivnosti.<br>- Jer mi studije omogućavaju da i dalje učim o mnogim stvarima koje me zanimaju.<br>- Zbog osećaja uzbuđenja koji doživljavam dok čitam o raznim zanimljivim temama.<br>- Jer mogu da doživim zadovoljstvo dok se trudim da budem uspešan/na na studijama.
Samoregulacija (SRQ-CZ)	*Navedite u kojoj meri svaka od sledećih stavki trenutno odgovara vašem izgledu. Molimo, koristite 1 „uopšte se ne slažem“ do 5 „potpuno se slažem“.*<br>- Obično pratim svoj napredak ka svojim ciljevima.<br>- Imam problema da se odlučim o stvarima.<br>- Lako se odvratim od svojih planova.<br>- Ne primećujem efekte svojih postupaka dok ne bude kasno.<br>- Teško mi je da vidim bilo šta korisno u promeni načina.<br>- Kada je u pitanju odluka o promeni, osećam se preopterećeno izborima.<br>- Imam problema da pratim stvari kada se odlučim da nešto uradim.<br>- Izgleda da ne učim na svojim greškama.<br>- Mogu da smislim mnogo načina da se promenim, ali mi je teško da odlučim koji da koristim.<br>- Mogu da se držim plana koji dobro funkcioniše.<br>- Obično samo jednom moram da pogrešim da bih iz nje naučio.<br>- Imam lične standarde i trudim se da ih ispunim.

	- Nametnuo sam se.<br>- Čim vidim problem ili izazov, počinjem da tražim moguća rešenja.<br>- Teško postavljam sebi ciljeve.<br>- Kada pokušavam nešto da promenim, mnogo obraćam pažnju na to kako mi ide.<br>- Čim vidim da stvari ne idu kako treba, želim da uradim nešto po tom pitanju.<br>- Obično postoji više načina da se nešto postigne.<br>- Imam pravila kojih se držim bez obzira na sve.<br>- Obično mogu da nađem nekoliko različitih mogućnosti kada želim da se promenim<br>- Često ne primetim šta radim dok mi neko ne skrene pažnju.<br>- Obično vidim potrebu da se promenim pre drugih.<br>- Dobar sam u pronalaženju različitih načina da dobijem ono što želim.<br>- Obično razmislim pre nego što reagujem.<br>- Učim na svojim greškama.<br>- Znam kako želim da budem.<br>- Brzo odustajem.
**Slovak version**	
Pohlavie	*Si ty?*<br>- Muž<br>- Žena
Vek	*Koľko máš rokov?*<br>- Presný vek vo formáte RRRR (roky)
Vzdelávanie	*Aké je vaše najvyššie dosiahnuté vzdelanie?*<br>- Základné alebo nedokončené vzdelanie<br>- Odborná alebo stredná škola bez maturity<br>- Odborné vzdelanie s maturitou<br>- Stredná škola s maturitou<br>- Gymnázium<br>- Vysoká škola – bakalárske štúdium<br>- Vysoká škola – magisterský stupeň<br>- Vysoká škola – doktorandský titul
Vnútorná motivácia (AMS-28)	*Pomocou nižšie uvedenej stupnice uveďte, do akej miery každá z nasledujúcich položiek v súčasnosti zodpovedá jednému z dôvodov, prečo sa zúčastňujete ďalšieho vzdelávania. Prosím, použite 1 „vôbec nezodpovedá“ až 7 „presne zodpovedá“.*

	- Pretože ma uspokojuje učenie sa nových vecí.<br>- Pretože mám dobrý pocit, keď sa môžem o svoje myšlienky podeliť s druhými.<br>- Pre pocit, ktorý zažívam, keď počas učenia prekonávam sám seba.<br>- Pre potešenie, ktoré mám z objavovania vecí, ktoré som predtým nepoznal(a).<br>- Pretože ma uspokojuje, keď si prečítam niečo zaujímavé.<br>- Pretože zažívam pocit uspokojenia, keď sa mi darí dosahovať moje osobné ciele.<br>- Pretože, keď študujem niečo zaujímavé, vyvolá to vo mne pozitívne pocity.<br>- Pretože mi prináša uspokojenie, keď sa zoznámim so zaujímavými myšlienkami.<br>- Pretože cítim uspokojenie, keď sa mi darí plniť náročné školské úlohy.<br>- Pretože mi štúdium umožňuje pokračovať v učení sa mnohým novým veciam, ktoré ma zaujímajú.<br>- Pre pocit uspokojenia, ktorý zažívam, keď sa dozvedám o zaujímavých veciach.<br>- Pretože mi univerzitné vzdelávanie umožní získať skúsenosti a pocit osobného uspokojenia pri mojom seba-rozvoji.
Samoregulácia (SRQ-CZ)	*Uveďte, do akej miery každá z nasledujúcich položiek v súčasnosti zodpovedá vášmu správaniu. Použite 1 „úplne nesúhlasím“ až po 5 „úplne súhlasím“.*<br>- Zvyčajne sledujem svoj pokrok smerom k svojim cieľom.<br>- Mám problém rozhodnúť sa o veciach.<br>- Ľahko sa nechám odviesť od svojich plánov.<br>- Nevšimnem si dôsledky svojich činov, kým nie je neskoro.<br>- Je pre mňa ťažké vidieť niečo užitočné na zmene môjho spôsobu života.<br>- Keď príde na rozhodnutie o zmene, cítim sa zavalený výberom.<br>- Mám problém dokončiť veci, keď som sa rozhodol niečo urobiť.<br>- Nezdá sa mi, že by som sa poučil zo svojich chýb.<br>- Môžem prísť na veľa spôsobov, ako sa zmeniť, ale je pre mňa ťažké rozhodnúť sa, ktorý použiť.<br>- Môžem sa držať plánu, ktorý funguje dobre.<br>- Väčšinou musím urobiť chybu len raz, aby som sa z nej poučil.<br>- Mám osobné štandardy a snažím sa ich dodržiavať.<br>- Som nastavený na svoje cesty.<br>- Hneď ako vidím problém alebo výzvu, začnem hľadať možné riešenia.<br>- Ťažko si stanovujem ciele.<br>- Keď sa snažím niečo zmeniť, dávam si veľký pozor na to, ako na tom som.<br>- Hneď ako vidím, že veci nejdú správne, chcem s tým niečo urobiť.<br>- Zvyčajne existuje viac spôsobov, ako niečo dosiahnuť.<br>- Mám pravidlá, ktorých sa držím, nech sa deje čokoľvek.

	- Keď sa chcem zmeniť, zvyčajne nájdem niekoľko rôznych možností<br>- Často si nevšimnem, čo robím, kým ma na to niekto neupozorní.<br>- Zvyčajne vidím potrebu zmeniť sa skôr, ako to urobia ostatní.<br>- Som dobrý v hľadaní rôznych spôsobov, ako dostať to, čo chcem.<br>- Väčšinou premýšľam, kým niečo urobím.<br>- Učím sa na vlastných chybách.<br>- Viem, aký chcem byť.<br>- Rýchlo sa vzdávam.
**Czech version**	
Pohlaví	*Jste?*<br>- Muž<br>- Žena
Věk	*Kolik je vám let?*<br>- Přesný věk ve formátu RRRR (roky)
Vzdělání	*Jaké je Vaše nejvyšší dosažené vzdělání?*<br>- Základní nebo nedokončené vzdělání<br>- Odborná nebo střední škola bez maturity<br>- Profesní s maturitou - Střední škola s maturitou<br>- Gymnázium<br>- Vysoká škola – bakalářský titul<br>- Vysoká škola – magisterské studium<br>- Vysoká škola – doktorský titul
Vnitřní motivace (AMS-28)	*Pomocí níže uvedené stupnice uveďte, do jaké míry každá z následujících položek v současnosti odpovídá jednomu z důvodů, proč se účastníte dalšího vzdělávání. Prosím, použijte 1 „vůbec neodpovídá“ až 7 „přesně odpovídá“.*<br>- Protože mne uspokojuje učení se nových věcí.<br>- Protože mám dobrý pocit, když se mohu o své myšlenky podělit s druhými.<br>- Pro pocit, který zažívám, když během učení překonávám sám sebe.<br>- Pro potěchu, kterou mám z objevování věcí, které jsem dříve nevěděl(a).<br>- Protože mne uspokojuje, když si přečtu něco zajímavého.<br>- Protože zažívám pocit uspokojení, když se mi daří dosahovat mých osobních cílů.<br>- Protože, když studuji něco zajímavého, vyvolá to ve mne pozitivní pocity.<br>- Protože mi přináší uspokojení, když se seznámím se zajímavými myšlenkami.

	- Protože cítím uspokojení, když se mi daří plnit obtížné školní úkoly.<br>- Protože mi studium umožňuje pokračovat v učení se mnoha novým věcem, které mne zajímají.<br>- Pro pocit uspokojení, který zažívám, když se dozvídám o zajímavých věcech.<br>- Protože mi univerzitní vzdělávání umožní získat zkušenosti a pocit osobního uspokojení při mém sebe-rozvoji.
Autoregulace (SRQ-CZ)	*Uveďte, do jaké míry každá z následujících položek v současnosti odpovídá vašemu chování. Použijte 1 „zcela nesouhlasím“ až po 5 „zcela souhlasím“.*<br>- Obvykle sleduji svůj pokrok směrem ke svým cílům.<br>- Mám problém rozhodnout se o věcech.<br>- Snadno se nechám odvést od svých plánů.<br>- Nevšimnu si důsledků svých činů, dokud není pozdě.<br>- Je pro mě těžké vidět něco užitečného vedoucí ke změně mého způsobu života.<br>- Když přijde na rozhodnutí o změně, cítím se zavalen(a) možnosti výběru.<br>- Mám problém dokončit věci, když jsem se rozhodl(a) něco udělat.<br>- Nezdá se mi, že bych se poučil ze svých chyb.<br>- Mohu přijít na mnoho způsobů, jak se změnit, ale je pro mě obtížné rozhodnout se, který použít.<br>- Mohu se držet plánu, který funguje dobře.<br>- Většinou musím udělat chybu jen jednou, abych se z ní poučil(a).<br>- Mám osobní standardy a snažím se je dodržovat.<br>- Jsem nastaven(a) na své cíle.<br>- Jakmile vidím problém nebo výzvu, začnu hledat možná řešení.<br>- Těžko si stanovuji cíle.<br>- Když se snažím něco změnit, dávám si velký pozor na to, jak na tom jsem.<br>- Jakmile vidím, že věci nejdou správně, chci s tím něco udělat.<br>- Obvykle existuje více způsobů, jak něčeho dosáhnout.<br>- Mám pravidla, kterých se držím, ať se děje cokoliv.<br>- Když se chci změnit, obvykle najdu několik různých možností<br>- Často si nevšimnu, co dělám, dokud mě na to někdo neupozorní.<br>- Obvykle vidím potřebu změnit se dříve, než to udělají ostatní.<br>- Jsem dobrý(á) v hledání různých způsobů, jak dostat to, co chci.<br>- Většinou přemýšlím, než něco udělám.<br>- Učím se na vlastních chybách.<br>- Vím, jaký(á) chci být.<br>- Rychle se vzdávám.
